# A Five-Year Trend Analysis of Antibacterial Resistance Patterns Among Non-fermenting Gram-Negative Bacilli: A Retrospective Study From the ICU Settings of a Tertiary Care Hospital

**DOI:** 10.7759/cureus.70375

**Published:** 2024-09-28

**Authors:** Rajesh K Dash, Ipsa Mohapatra, Nipa Singh, Dipti Pattnaik, Subhra Snigdha Panda, Shradha Smriti, Kalpana Mund, Preety Mishra, Soumya Nayak, Swarupa Mohapatra

**Affiliations:** 1 Department of Microbiology, Kalinga Institute of Medical Sciences, Bhubaneswar, IND; 2 Department of Community Medicine, Kalinga Institute of Medical Sciences, Bhubaneswar, IND

**Keywords:** antibiotic resistance trend, icu, mdr bacteria, non-fermenting gram negative bacilli, prevalence

## Abstract

Introduction: Non-fermenting gram-negative bacilli (NFGNB) infections have emerged as a serious health concern in ICUs. Multi-drug resistant (MDR) strains of NFGNB can evolve by acquiring resistance genes to at least one agent in three or more antibacterial categories. This study aimed to analyse the prevalence of NFGNB, the distribution of MDR strains, and antibiotic resistance trends of NFGNB in different ICUs of a tertiary care hospital over a period of five years.

Materials and methods: This retrospective study was conducted in a tertiary care teaching hospital in eastern India, including a total of 20,256 samples received from various ICUs over five years. Data retrieved from the Laboratory Information System (LIS) of the hospital, and repetitive isolates from the same patients, were excluded. All samples were processed according to standard microbiological protocols by automated systems. Data were entered into a Microsoft Excel spreadsheet (Microsoft® Corp., Redmond, WA, USA), analysed using Epi Info software, and presented using descriptive statistics. Chi-square and Fisher’s exact tests (where appropriate) were used as tests of significance, with a p-value of <0.05 considered statistically significant.

Results: A total of 18,032 culture-positive samples out of 20,256 samples showed growth of 18,659 bacteria. Out of these, 952 isolates were NFGNB. The prevalence of NFGNB was found to be 5.10% among all isolated bacteria. The predominant sources were respiratory samples (37.3%). *Acinetobacter* spp. emerged as the most prevalent NFGNB (46.5%), followed by *Pseudomonas* spp. (31%) and *Burkholderia* spp. (14.3%). Among the NFGNB isolates, 61.76% exhibited MDR, with the highest prevalence of MDR strains seen in *Elizabethkingia* spp. (94.7%). Among the most prevalent NFGNB, *Acinetobacter* spp., 64.8% were MDR strains. Trend analysis of antibiotic resistance patterns of *Acinetobacter* spp. indicated a substantial increase for trimethoprim-sulfamethoxazole by 18.5%, minocycline (44.4%), amikacin (20.4%), and ceftazidime (7.4%), whereas there was a reduced trend in resistance to carbapenems (6.5%), ciprofloxacin (4.7%), and cefepime (3.7%) over five years. In *Pseudomonas* spp., resistance to meropenem increased by 17.4%, and for ceftazidime (11.8%), amikacin (10.6%), and piperacillin-tazobactam (7.9%), whereas resistance to aztreonam diminished by 13.9%. *Burkholderia* spp. exhibited a 23.5% escalation in resistance to meropenem and ceftazidime (5.9%), while resistance to levofloxacin experienced a decrease of 30.2%.

Conclusions: The study showed the prevalence of various NFGNB as 5.10% in ICU settings, with *Acinetobacter* spp. (46.5%) being the most common isolated bacteria. Notably, 61.76% of the isolates were MDR. Antibiotic trend analysis over five years showed increasing resistance of *Acinetobacter* spp. to trimethoprim-sulfamethoxazole, minocycline, and ceftazidime, with improved susceptibility for carbapenems, ciprofloxacin, and cefepime. *Pseudomonas* spp. showed increased susceptibility to aztreonam and rising resistance for meropenem, piperacillin-tazobactam, ceftazidime, and amikacin. In Burkholderia spp., there was increased susceptibility to levofloxacin and rising resistance to meropenem and ceftazidime. These findings focus on the need for vigilant antibiotic stewardship, with the adoption of appropriate infection prevention and control practices to restrict the emergence and spread of MDR NFGNB infections in ICU settings of hospitals.

## Introduction

Non-fermenting gram-negative bacilli (NFGNB) are obligatory aerobic and non-sporing bacteria, which use oxidative pathways for the breakdown of carbohydrates [1]. NFGNBs are ubiquitous in nature and can grow everywhere in healthcare settings, including various equipment, parenteral fluids, distilled water, and sinks, because of their intrinsic resistance to commonly used disinfectants [2].

*Pseudomonas* spp. are the predominant NFGNB isolated from patients, followed by *Acinetobacter* spp., *Burkholderia* spp., and *Stenotrophomonas maltophilia*, etc. [3]. Immunocompromised patients with HIV/AIDS, Type II diabetes, those taking corticosteroids, cancer patients undergoing chemotherapy with weakened immune systems, patients on immunosuppressants in organ transplant cases, and those admitted to ICUs are at risk of developing healthcare infections due to NFGNB [4].

NFGNB can cause serious healthcare-associated infections, with a spectrum of diseases including urinary tract infections, pneumonia, septicaemia, meningitis, surgical site infections, and infections in skin and soft tissue [5]. In ICUs, an increasing trend of infections due to NFGNB, such as *Pseudomonas* spp. and *Acinetobacter* spp., has been observed, which may be attributed to the higher use of invasive devices and prolonged hospital stays [6]. Inadequate availability of data regarding empirical antibiotic treatment for NFGNB infections in recent times has made these pathogens significant in hospital care settings [7]. NFGNB constitute about 12%-16% of all bacterial isolates cultured from various clinical samples [8].

Growing antibacterial resistance among NFGNB, particularly *Acinetobacter* spp., has become a global health concern. According to the Centers for Disease Control and Prevention (CDC, USA), carbapenem-resistant *Acinetobacter* spp. is regarded as the “most urgent antimicrobial resistance (AMR) threat,” while multi-drug resistant (MDR) *Pseudomonas aeruginosa* is considered a “serious threat” [9].

MDR strains of NFGNB infections have become a matter of concern among patients [10]. MDR bacteria were defined as “acquired non-susceptibility to at least one agent in three or more antimicrobial categories” [11].

The emergence of NFGNB in ICUs and their increasing trend of resistance to commonly available antibiotics demand detail knowledge of prevalence of these bacteria and their antibiotic susceptibility pattern. The present study was planned to find out the prevalence of NFGNB causing infections in ICU settings, the distribution of MDR strains and the changing trend of antibiotic resistance of NFGNB over five years. The study also compared the rate of isolation of NFGNB among different age groups and gender (male and female) of the patients.

This study has been presented as a poster at the sixth National Conference and Workshop on Antimicrobial Stewardship Practices in India (ASPICON 2024), held at PGIMER, Chandigarh.

## Materials and methods

Setting and data collection

This is a retrospective data-based study, where data was collected from the Laboratory Information System (LIS) for the samples enrolled during the study period (January 1, 2019-December 31, 2023) in the Department of Microbiology. Data was entered into a Microsoft Excel spreadsheet (Microsoft® Corp., Redmond, WA, USA) for further analysis. This data included the patient’s age, sex, bacteria name, year of isolation, and antibiotic sensitivity of bacterial isolates. All recorded data regarding samples, such as blood, urine, tissue, pus, and respiratory samples (sputum, broncho-alveolar lavage, endotracheal aspirate), as well as fluid samples (cerebrospinal fluid, ascitic fluid, pleural fluid, etc.), received in the laboratory from patients admitted to different ICUs - i.e., the main ICU, medicine ICU (MICU), neuro ICU, paediatric ICU (PICU), neonatal ICU (NICU), and cardiac ICU (CICU) - were included. This study was approved by the Institutional Ethics Committee (KIIT/KIMS/IEC/1425/2023). Duplicate isolates of the same patient were excluded from the analysis, including only the first isolate of each patient.

Sample collection and processing

In our laboratory, an automated blood culture system (BacT/ALERT 3D; bioMérieux, Marcy-l'Étoile, France) was used for blood and body fluid cultures. These samples were collected before the administration of antibiotics in blood culture bottles (BacT/ALERT PF plus for paediatric patients and BacT/ALERT FA plus for adults), after adopting proper aseptic measures and incubated in the automated system for five days (for negative cultures) or until the detection of positive growth, as per the manufacturer's instructions. After the indication of positive growth by the automated systems, the bottles were removed, and subcultures were performed. The blood and body fluid samples were subcultured on blood agar (bioMérieux) and MacConkey agar (HiMedia, Mumbai, India). Other samples were processed according to standard microbiological protocols [12]. The plates were incubated at 37°C for 48 hours and monitored daily for bacterial growth.

Bacterial identification and antibacterial susceptibility testing

The identification and antibacterial susceptibility testing were done using the VITEK 2 compact system (bioMérieux). The antibiotic sensitivity results were interpreted according to the Clinical and Laboratory Standards Institute (CLSI) guidelines [13].

Statistical analysis

Data entered in the Microsoft Excel spreadsheet was analysed using Epi Info statistical software (version 7.2.3.1). All categorical data were presented using frequencies and percentages. Continuous data were presented as means with standard deviations and medians with interquartile ranges, as applicable. Bacterial isolates numbering below 30 were not considered for the calculation of the prevalence of MDR bacteria and the trend of antibiotic resistance. Chi-square tests and Fisher’s exact tests were used as tests of significance (where appropriate). A p-value of <0.05 was considered statistically significant.

## Results

Between January 1, 2019, and December 31, 2023, a total of 20,256 samples were received from different ICUs for culture. Out of the total, 18,032 culture-positive samples were collected; 17,405 samples isolated a single type of bacterial growth, and 627 samples isolated two types of bacteria. A total of 18,659 bacteria were isolated and processed for identification and antibiotic sensitivity testing in the laboratory. Of these, 56.87% (10,612/18,659) were gram-negative bacteria, and 43.13% (8,047/18,659) were gram-positive bacteria. Among all the bacteria isolated, the prevalence of NFGNB was found to be 5.10% (952/18,659). Male patients showed a higher isolation rate of NFGNB (72.2%, 687/952) than female patients, who had a rate of 27.8% (265/952).

Out of 952 NFGNB isolates included in the current analysis, 443 (46.5%) were *Acinetobacter* spp., 295 (31.0%) were *Pseudomonas* spp., 136 (14.3%) were *Burkholderia* spp., 38 (4.0%) were *Elizabethkingia* spp., 29 (3.0%) were *Sphingomonas* spp., nine (0.9%) were *Stenotrophomonas* spp., and two (0.2%) were *Chryseobacterium* spp.

**Figure 1:**
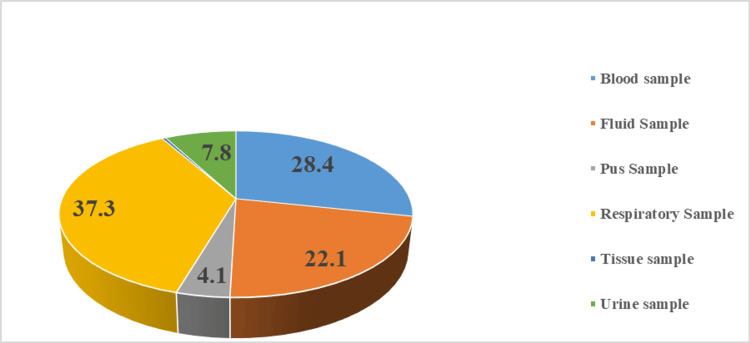
Sample wise distribution (in percentage) of NFGNB isolates (N = 952) NFGNB: Non-fermenting gram-negative bacilli

The majority of isolates obtained were recovered from respiratory samples (37.3%, 355/952), followed by blood samples (28.4%, 270/952) and fluid samples (22.1%, 210/952) (Figure 1).

Table 1 shows the distribution of NFGNB in different ICUs. *Acinetobacter* spp. were the most predominant isolates in all ICUs, except in the CICU, where *Pseudomonas* spp. were the predominant bacteria.

**Table 1:** Five-year prevalence of the NFGNB in various ICUs (N = 952) CICU: Cardiology intensive care unit; MICU: Medicine intensive care unit; NICU: Neonatal intensive care unit; PICU: Paediatric intensive care unit; NFGNB: Non-fermenting gram-negative bacilli

Bacteria	CICU (n = 6)	Main ICU (n = 419)	MICU (n = 226)	Neuro ICU (n = 223)	NICU (n = 39)	PICU (n = 39)
	N (%)	N (%)	N (%)	N (%)	N (%)	N (%)
*Acinetobacter* spp.	2 (33.3)	192 (45.8)	113 (50)	99 (44.4)	18 (46.2)	19 (48.7)
*Burkholderia* spp.	0 (0)	72 (17.2)	28 (12.4)	22 (9.9)	6 (15.4)	8 (20)
*Chryseobacterium* spp.	0	2 (0.5)	0	0	0	0
*Elizabethkingia* spp.	0 (0)	20 (4.8)	11 (4.9)	5 (2.2)	2 (5.1)	0 (0)
*Pseudomonas* spp.	3 (50)	122 (29.1)	69 (30.5)	87 (39)	4 (10.3)	10 (25.6)
*Sphingomonas* spp.	1 (16.7)	7 (1.7)	5 (2.2)	6 (2.7)	9 (23.1)	1 (2.6)
*Stenotrophomonas* spp.	0 (0)	4 (1)	0 (0)	4 (1.8)	0 (0)	1 (2.6)

**Figure 2:**
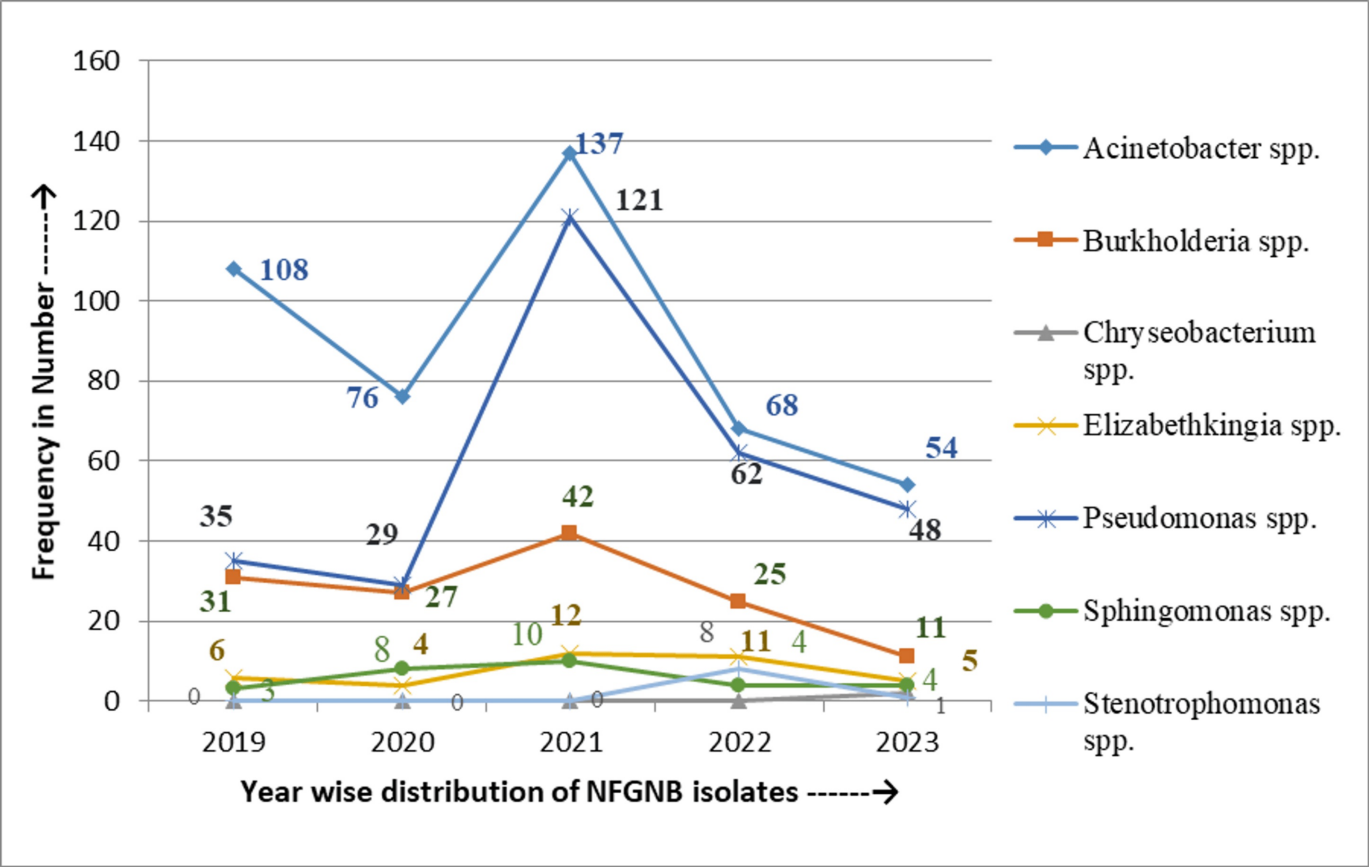
Five-year prevalence trends of the NFGNB isolates from the ICUs The five-year (2019-2023) prevalence of NFGNB in numbers of bacteria isolated year-wise NFGNB: Non-fermenting gram-negative bacilli

Figure 2 shows the five-year prevalence of NFGNB in various ICUs in terms of the number of bacteria isolated year-wise. From 2019 to 2023, *Acinetobacter* spp. was consistently the most prevalent NFGNB among ICU patients, varying from 68 (38.2%, 68/178) in 2022 to 108 (59.0%, 108/183) in 2019. *Pseudomonas* spp. were the most frequently isolated bacteria in 2021 and 2022, accounting for 121 (37.6%, 121/322) and 62 (34.8%, 62/178), respectively. Meanwhile, *Burkholderia* spp. showed maximum isolation, up to 27 (18.8%, 27/144) in 2020.

**Figure 3:**
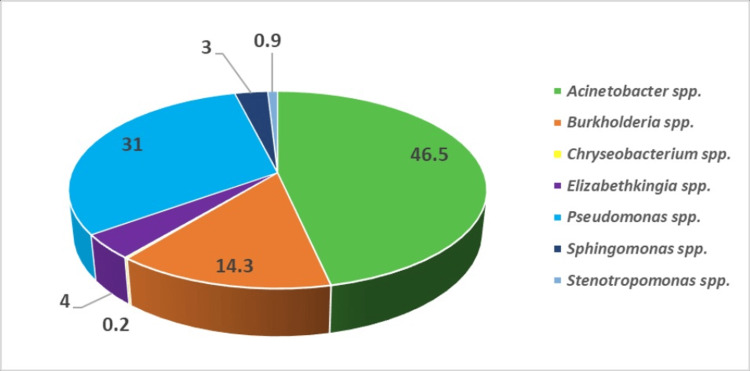
Distribution (in percentage) of NFGNB isolates (N = 952) NFGNB: Non-fermenting gram-negative bacilli

The most frequently isolated bacteria were *Acinetobacter* spp. (46.5%, 443/952), followed by *Pseudomonas* spp. (31%, 295/952) and *Burkholderia* spp. (14.3%, 136/952) (Figure 3).

A total of 952 NFGNB were isolated from 18,032 culture-positive clinical samples, accounting for an isolation rate of 5.23% (952/18,032) from the culture-positive samples. Of the total NFGNB, 588 (61.76%, 588/952) were MDR strains. The majority of MDR NFGNB isolated were from respiratory samples (40.6%, 239/588), followed by blood samples (26.9%, 158/588), fluid samples (18.7%, 110/588), urine samples (8.7%, 51/588), and pus samples (4.4%, 4/588), respectively. The majority of MDR NFGNB were isolated from patients in the age group of more than 64 years (32%, 188/588), while the least were from the age group of under one month (3.1%, 18/588).

Among the NFGNB, the highest rate of MDR strains was found in *Elizabethkingia* spp. (94.7%, 36/38), followed by *Burkholderia* spp. (73.5%, 100/136) and *Acinetobacter* spp. (64.8%, 287/443) (Table 2).

**Table 2:** Distribution of MDR bacteria in NFGNB MDR: Multi-drug resistance; NFGNB: Non-fermenting gram-negative bacilli

Number of NFGNB isolates (N)	Number of MDR bacteria, n (%)
*Acinetobacter* spp. (443)	287 (64.8%)
*Burkholderia* spp. (136)	100 (73.5%)
*Chryseobacterium* spp. (2)	2 (100.0%)
*Elizabethkingia* spp. (38)	36 (94.7%)
*Pseudomonas* spp. (295)	156 (52.9%)
*Sphingomonas* spp. (29)	7 (24.1%)
*Stenotrophomonas* spp. (9)	0 (0.0%)
Total (N = 952)	588 (61.76%)

**Figure 4:**
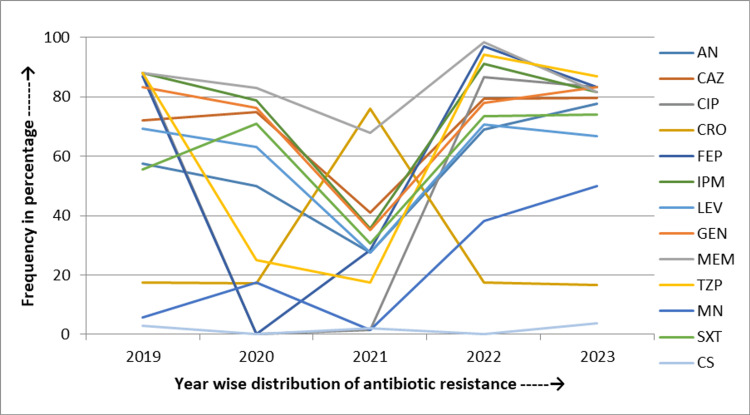
Five-year linear trend of antibiotic resistance of Acinetobacter spp. AN: Amikacin; CAZ: Ceftazidime; CIP: Ciprofloxacin; FEP: Cefepime; CRO: Ceftriaxone; IPM: Imipenem; LEV: Levofloxacin; MEM: Meropenem; GEN: Gentamicin; TZP: Piperacillin and tazobactam; MN: Minocycline; SXT: Trimethoprim-sulfamethoxazole

During the five years of the study, *Acinetobacter* spp. exhibited a sharp rise in resistance rates for trimethoprim-sulfamethoxazole by 18.5% (from 55.6% to 74.1%), minocycline by 44.4% (from 5.6% to 50%), and amikacin by 20.4% (from 57.4% to 77.8%). In addition, increasing resistance was seen for ceftazidime by 7.4% (from 72.2% to 79.6%). Conversely, resistance rates decreased for carbapenem by 6.5% (from 88% to 81.5%), ciprofloxacin by 4.7% (from 88% to 83.3%), and cefepime by 3.7% (from 87% to 83.3%) over the same period (Figure 4).

**Figure 5:**
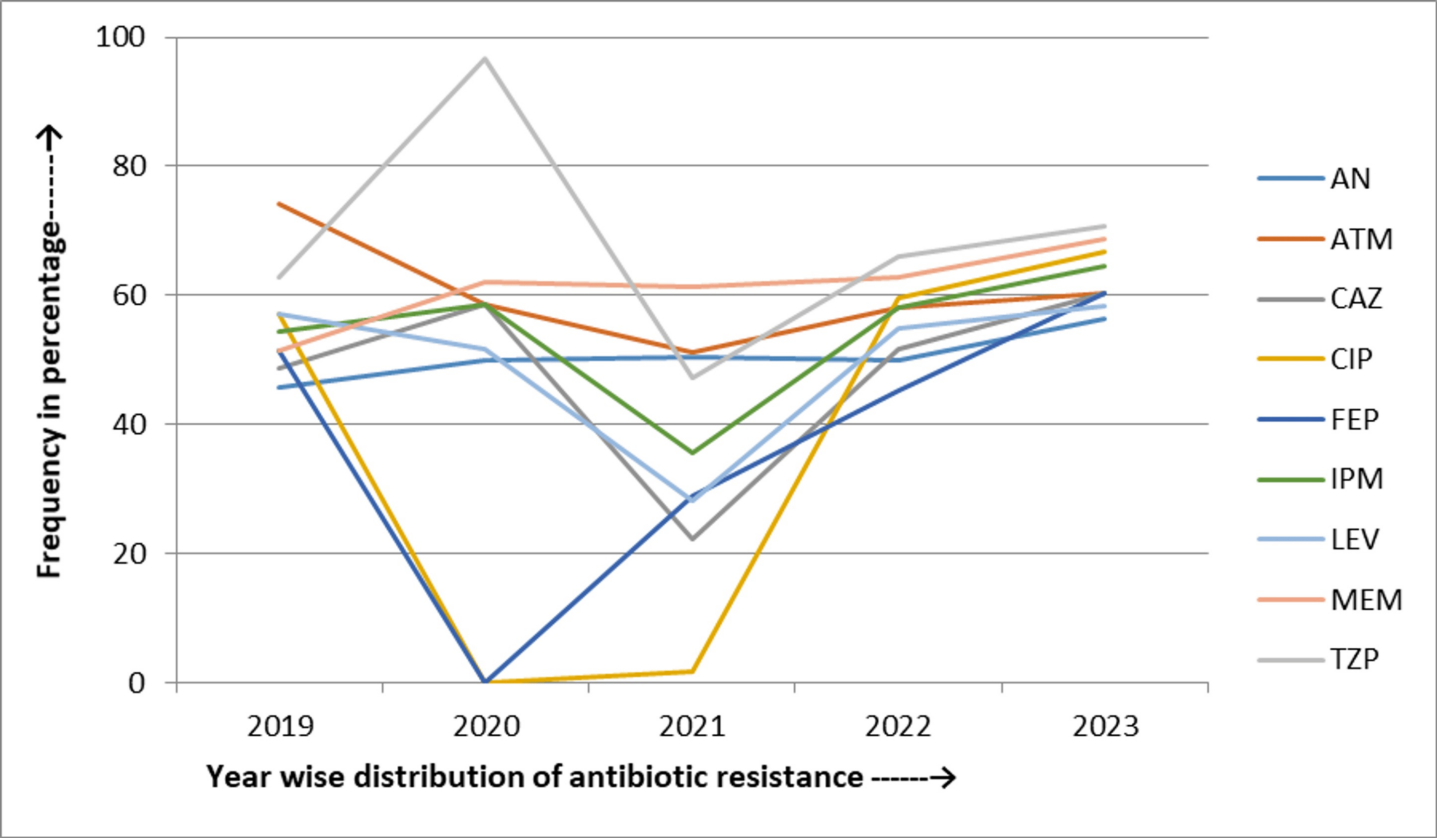
Five-year linear trend of antibiotic resistance of Pseudomonas spp. AN: Amikacin; ATM: Aztreonam; CAZ: Ceftazidime; FEP: Cefepime; CIP: Ciprofloxacin; IPM: Imipenem; LEV: Levofloxacin; MEM: Meropenem; TZP: Piperacillin and tazobactam

*Pseudomonas* spp. exhibited a significant increase in resistance rates from 2019 to 2023, with meropenem resistance rising by 17.4% (from 51.4% to 68.8%), ceftazidime by 11.8% (from 48.6% to 60.4%), amikacin by 10.6% (from 45.7% to 56.3%), ciprofloxacin by 9.6% (from 57.1% to 66.7%), cefepime by 9% (from 51.4% to 60.4%), and piperacillin-tazobactam by 7.9% (from 62.9% to 70.8%). In contrast, the resistance rate to aztreonam decreased by 13.9% (from 74.3% to 60.4%) during the same period (Figure 5).

**Figure 6:**
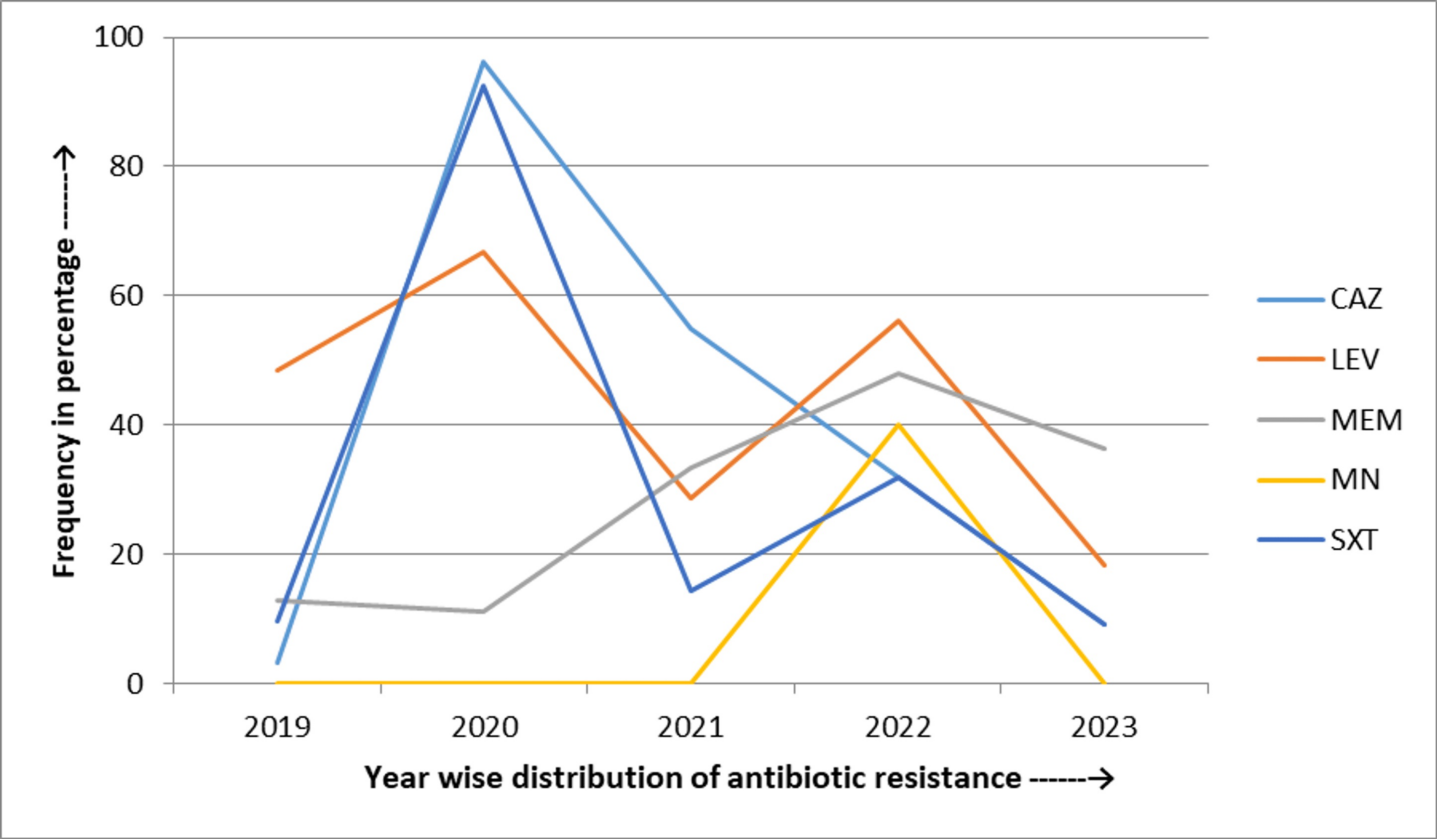
Five-year linear trend of antibiotic resistance of Burkholderia spp. CAZ: Ceftazidime; LEV: Levofloxacin; MEM: Meropenem; MN: Minocycline; SXT: Trimethoprim-sulfamethoxazole

In *Burkholderia* spp., ceftazidime resistance spiked dramatically by 5.9% (from 3.2% to 9.1%), and meropenem resistance increased by about 23.5% (from 12.9% to 36.4%) during these five years, while resistance to levofloxacin decreased by 30.2% (from 48.4% to 18.2%) (Figure 6).

**Figure 7:**
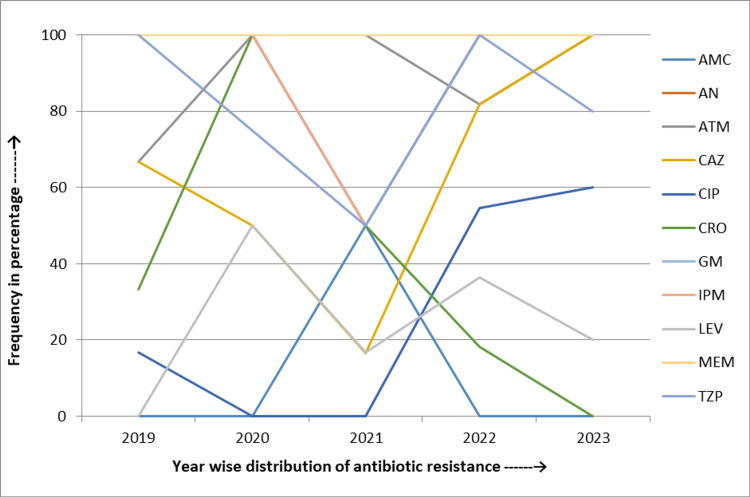
Five-year linear trend of antibiotic resistance of Elizabethkingia spp. AMC: Amoxicillin/clavulanate; AN: Amikacin; ATM: Aztreonam; CAZ: Ceftazidime; CIP: Ciprofloxacin; CRO: Ceftriaxone; GM: Gentamicin; IPM: Imipenem; LEV: Levofloxacin; MEM: Meropenem; TZP: Piperacillin and tazobactam

*Elizabethkingia* spp. showed consistently high resistance to multiple antibiotics. Amikacin and meropenem resistance remained at 100% throughout the study period. Resistance to ceftazidime and imipenem was high, peaking at 100% in 2023. Resistance to ciprofloxacin increased to 60% in 2023, aztreonam resistance rose from 66.7% to 100% (a 33.3% increase), and gentamicin resistance remained consistently high (Figure 7).

The mean age was 51.77 ± 22.14 SD, with a range of four months to 95 years. The median age was 55.15 years (IQR, 39 to 68 years), and 72.06% of the study population were males.

The distribution of NFGNB-causing infections in various ICU patients varied with the different bacterial isolates and age groups of the patients. *Acinetobacter* spp. were the most commonly isolated bacteria in elderly patients (>64 years), accounting for 36.8%, with a significant p-value of 0.04. *Burkholderia* spp. were predominantly obtained from patients aged between 49 and 64 years (33.1%), followed by the age group of 33-48 years (25%), with a p-value of 0.04 (Table 3).

**Table 3:** Age and gender wise distribution of different types of NFGNB *using Chi-square test; **using Fisher exact test NFGNB: Non-fermenting gram-negative bacilli

Bacteria	*Acinetobacter* spp. (n = 443)	*Pseudomonas* spp. (n = 295)	*Burkholderia* spp. (n = 136)	*Chryseobacterium* spp. (n = 2)	*Elizabethkingia* spp. (n = 38)	*Sphingomonas* spp. (n = 29)	*Stenotrophomonas* spp. (n = 9)
	N (%)	N (%)	N (%)	N (%)	N (%)	N (%)	N (%)
Age-group							
<1 month (n = 40)	19 (4.3)	4 (1.4)	6 (4.4)	0 (0)	2 (5.3)	9 (31)	0 (0)
1 months-16 years (n = 39)	18 (4.1)	10 (3.4)	8 (5.9)	0 (0)	0 (0)	1 (3.4)	1 (11.1)
17-32 years (n = 105)	33 (7.4)	55 (18.6)	11 (8.1)	0 (0)	0 (0)	2 (6.9)	0 (0)
33-48 years (n = 185)	86 (19.4)	43 (14.6)	34 (25)	0 (0)	7 (18.4)	4 (13.8)	1 (11.1)
49-64 years (n = 293)	124 (28)	91 (30.8)	45 (33.1)	1 (50)	9 (23.7)	7 (24.1)	7 (77.8)
>64 years (n = 331)	163 (36.8)	92 (31.2)	32 (23.5)	1 (50)	20 (52.6)	6 (20.7)	0 (0)
p-value	0.04*	3.54*	0.04*	0.94*	0.07*	6.2*	0.02*
Gender							
Male (n = 687)	332 (74.9)	205 (69.5)	98 (0)	2 (100)	25 (65.8)	17 (58.6)	8 (88.9)
Female (n = 265)	111 (25.1)	90 (30.5)	38 (27.9)	0 (0)	13 (34.2)	12 (41.4)	1 (11.1)
p-value	0.08*	0.25*	0.92*	0.92**	0.48*	0.15*	0.45*

## Discussion

Infections among patients admitted to ICUs have emerged as a concern, despite the awareness and proper care taken by healthcare workers [14]. Previously, gram-negative bacteria, predominantly from the “*Enterobacteriaceae*” family, have taken the upper hand in causing infections in hospital settings. NFGNB were considered pathogens of less significance because of their ubiquitous nature in the hospital environment [12]. In the current scenario, NFGNB has become a therapeutic challenge for treating physicians due to the emergence of MDR strains and their propensity to affect critically ill and immunocompromised groups, causing opportunistic infections. These factors are increasing patients’ morbidity, mortality, and the cost of treatment [5].

Various literature suggests emerging drug resistance in different types of NFGNB, such as *Acinetobacter* spp., *Pseudomonas* spp., *Burkholderia* spp., and *Elizabethkingia* spp. [10]. The bacteria may have intrinsic resistance mechanisms or may undergo gene alteration due to mutations or through plasmids, or they may acquire drug-resistance genes in the course of treatment with a particular group of antibiotics [1,3,15]. The different mechanisms of resistance include altered membrane permeability, altered target sites, efflux pumps, loss through porins, and production of drug-inactivating enzymes [15].

The isolation rate of NFGNB among all the culture-positive samples was 5.23% in the present study. The prevalence of NFGNB was 5.10% among all the bacteria obtained from the culture-positive samples. The isolation rate of NFGNB was higher in male patients (72.2%) than in female patients (27.8%). In other studies, such as those conducted by Grewal et al. in India, the isolation rate of NFGNB was reported as 11.6%; Rit et al. reported it as 12.8%; and Benachinmardi et al. reported it as 10% [8,16,17]. This difference may be explained by the effective infection control practices carried out in our setting, which likely reduced the transmission of NFGNB from environmental sources.

In our study, a majority of NFGNB isolates were obtained from respiratory samples (37.3%), followed by blood samples (28.4%) and fluid samples (22.1%). Our results are in congruence with the observations made by Nautiyal et al., who found respiratory samples (42.3%) to be the major source of NFGNB [18]. In contrast, Bhatnagar et al. found pus samples to be the primary source of NFGNB isolates (49.2%), while sputum samples were the second most frequent source of NFGNB (19.8%) [19]. Additionally, studies by Rit et al. (27.86%) and Gokale and Metgud (58.4%) demonstrated that pus samples were a primary source of NFGNB [16,20]. These disparities may stem from variations in the types of healthcare-associated infections prevalent in each respective study. For example, our findings and those of Nautiyal et al. may indicate that respiratory infections in the ICUs are the predominant infections [18].

There are substantial differences in the distribution of bacterial species among various ICUs. *Acinetobacter* spp. was the most common bacteria found in the MICU (50%), followed by the main ICU (45.8%). In contrast, a study done by Moolchandani et al. showed *Acinetobacter* spp. (21.7%) to be the most frequently isolated bacteria in the adult ICU. *Pseudomonas* spp. predominance was found in the PICU (18.8%) [21]. This variation in the distribution of bacteria may be explained by factors such as patients’ conditions - like chronic illness, immunocompromised states, invasive procedures, and infection control practices - along with the environmental factors of the different ICUs.

*Acinetobacter* spp. and *Pseudomonas* spp. were the most frequently isolated bacteria over the five-year study period. *Acinetobacter* spp. peaked at 108 (59.0%, 108/183) in 2019 and decreased to 68 (38.2%, 68/178) in 2022, while *Pseudomonas* spp. reached its highest prevalence at 121 (37.6%, 121/322) in 2021. *Burkholderia* spp. reached its highest peak at 27 (18.8%, 27/144) in 2020, and no marked differences in the distribution of other bacteria were found. Another study by Ndzabandzaba et al. showed a decreasing isolation rate of *Acinetobacter* spp. (from 71% to 62%), whereas the total number of *Pseudomonas* spp. showed a significant increase (from 23% to 28%) over three years [22].

Among the total of 952 isolates of NFGNB, *Acinetobacter* spp. emerged as the predominant species (46.5%), followed by *Pseudomonas* spp. (31%) and *Burkholderia* spp. (14.3%). This distribution coincides with the findings of other authors, such as Yadav et al., who identified *Acinetobacter* spp. as the most common NFGNB, constituting 44%, followed by *Pseudomonas* spp. (40.1%). In the study by Nazir et al., *Acinetobacter* spp. exhibited a substantially higher prevalence (79.1%), followed by *Pseudomonas* spp. (9.1%) [5,23]. These disparities may be attributed to variations in local epidemiological factors, the distribution of NFGNB in the hospital environment, infection prevention and control practices, or characteristics of the study populations. The predominance of *Acinetobacter* spp. in our study can be explained by its prevalence in healthcare settings, particularly within ICUs. These bacteria have established themselves as successful pathogens in hospital settings, especially in ICUs, due to their well-adapted nature, capacity to form biofilms on medical equipment, bedding, and floors, and the emergence of MDR strains affecting immunocompromised patients [24].

The present study showed the isolation of MDR NFGNB to be 61.76%, compared to 78.21% as reported by Yadav et al. [5]. More judicious use of antibiotics and limiting the spread of resistant strains may help reduce MDR bacteria. Most MDR NFGNB in our study were from respiratory samples (40.6%), followed by blood samples (26.9%) and body fluids (18.7%). In contrast, Soni et al. found the majority from pus (45.50%) and blood samples (20.50%) [7]. The highest prevalence of MDR NFGNB in our study was among patients over 64 years (32%), with the lowest in infants under one month (3.1%). In the study by Soni et al., the maximum number of MDR NFGNB was found in the age group of 46-65 years, and the least in infants (0-1 years) [7].

The MDR rate for *Acinetobacter* spp., the most prevalent isolate in our investigation, was found to be 64.8%, which is rather high but lower than the rates reported by Shrestha et al. (96%) and Mishra et al. (95%) [25,26]. Judicious use of broad-spectrum antibiotics and proper infection control practices may be the reason for the lower MDR incidence in the present study. The maximum MDR strains isolated were from *Elizabethkingia* spp. (94.7%), followed by *Burkholderia* spp. (73.5%) and *Acinetobacter* spp. (64.8%). *Elizabethkingia* spp. mostly occur as common contaminants found in water sources or the surroundings of ICU settings and can acquire resistance genes through horizontal gene transfer, leading to the emergence of MDR strains [27].

From 2019 to 2023, *Acinetobacter* spp. exhibited increasing resistance to trimethoprim-sulfamethoxazole (18.35%) and amikacin (20.4%). Similarly, resistance rates decreased for ciprofloxacin (4.7%) and cefepime (3.7%). In a study by Kumari et al., they showed a slight increase in resistance to trimethoprim-sulfamethoxazole (1.75%) and a decrease in resistance to amikacin (12.3%) over five years. Maraki et al. reported a decreasing resistance trend for amikacin (33.9%) [28,29]. These variations could be attributed to local antibiotic usage, infection control practices, and regional resistance patterns. Our findings suggest a significant increasing trend of antibiotic resistance in *Acinetobacter* spp., highlighting the need for appropriate surveillance and tailored infection control measures.

In *Pseudomonas* spp., an increase in resistance rates was found for meropenem (17.4%), ceftazidime (11.8%), amikacin (10.6%), ciprofloxacin (9.6%), cefepime (9%), and piperacillin-tazobactam (7.9%). In contrast, the resistance rate decreased for aztreonam (13.9%) during the same period. Another study by Lyu et al. found a slightly increasing trend of resistance for ceftazidime (1.6%), amikacin (0.3%), and aztreonam (4.5%), along with a decreasing trend for meropenem (1.7%) [30]. The significant rise in resistance in our study could be due to the more frequent use of these antibiotics, especially in ICU settings.

Between 2019 and 2023, *Burkholderia* spp. in our study exhibited significant resistance fluctuations, particularly in ceftazidime, where resistance surged from 3.2% in 2019 to 96.3% in 2020, then dropped to 9.1% by 2023. In the case of meropenem, an increased resistance trend was observed (23.5%). Similarly, another study by Sethi et al. showed an increasing resistance trend for ceftazidime (18%) and a decrease in resistance for meropenem (10%) [31].

Our study identified 100% resistance to amikacin, ceftazidime, and carbapenems in *Elizabethkingia* spp., indicating limited treatment options. This is consistent with the findings of Comba et al., who reported resistance rates of 88.2% for amikacin, 99% for aztreonam, and 95.4% for ceftazidime [32]. The slightly lower resistance observed in Comba et al.'s study could be attributed to differences in sample size, bacterial strain variations, or environmental factors affecting resistance mechanisms.

Our study highlights the distribution of NFGNB in ICU patients, revealing that *Acinetobacter* spp. was most prevalent (36.8%), particularly in the elderly (>64 years). *Burkholderia* spp. (33.1%) was predominantly found in middle-aged patients (49-64 years). This distribution was also found to be statistically significant. *Pseudomonas* spp. showed a more even distribution across all age groups. In the study conducted by Sharma et al., the majority of *Acinetobacter* spp. were isolated from the older age group (61-70 years), followed by the 81-90 years age group. Yadav et al. and Grewal et al. found that most NFGNB isolates were obtained from male patients [5,8,33]. No such association of NFGNB distribution according to the gender of the patients could be established in the present study. These findings emphasize the need for targeted infection control in vulnerable age groups, particularly the elderly.

Limitations

We could not retrieve data for the evaluation of risk factors and patient outcomes in NFGNB infection cases due to the retrospective nature of the study. We also missed the opportunity to perform molecular characterization of antibiotic-resistant bacteria.

## Conclusions

NFGNB, once considered contaminants in hospital settings, are emerging as a serious cause of infections in ICU settings. The rise of MDR strains among NFGNB is limiting the available treatment options. This study has evaluated resistance trends of NFGNB in ICU settings over five years. The prevalence of NFGNB was found to be 5.10% among all the bacteria isolated. The most common NFGNB was *Acinetobacter* spp. (46.5%), followed by *Pseudomonas* spp. (31%) and *Burkholderia* spp. (14.3%), with 61.76% of these isolates identified as MDR. The highest MDR rates were observed in *Elizabethkingia* spp. (94.7%) and *Burkholderia* spp. (73.5%). During the study period, *Acinetobacter* spp. exhibited increasing resistance to trimethoprim-sulfamethoxazole, minocycline, and ceftazidime, with improved susceptibility to carbapenems, ciprofloxacin, and cefepime. *Pseudomonas* spp. showed increased susceptibility to aztreonam while exhibiting rising resistance to meropenem, piperacillin-tazobactam, ceftazidime, and amikacin. *Burkholderia* spp. demonstrated enhanced susceptibility to levofloxacin and rising resistance to meropenem and ceftazidime. *Elizabethkingia* spp. displayed increasing resistance to aztreonam and other antibiotics.

This study emphasizes the need to address the distribution and resistance patterns of NFGNB in different hospital care settings, particularly in critical care units. This will further help in formulating a proper antibiotic policy to combat NFGNB infections. Additionally, the spread of MDR strains of NFGNB can be curtailed through the proper implementation of appropriate infection prevention and control practices, antimicrobial stewardship, and continuous surveillance.
